# Magnetically Powered Biodegradable Microswimmers

**DOI:** 10.3390/mi11040404

**Published:** 2020-04-13

**Authors:** Ho Cheung Michael Sun, Pan Liao, Tanyong Wei, Li Zhang, Dong Sun

**Affiliations:** 1 King George V School, Hong Kong 999077, China; 19sunh3@kgv.hk; 2 Department of Biomedical Engineering, City University of Hong Kong, Hong Kong 999077, China; pliao7@cityu.edu.hk (P.L.); tanyong.wei@my.cityu.edu.hk (T.W.); 3 Department of Mechanical and Automation Engineering, The Chinese University of Hong Kong, Hong Kong 999077, China; lizhang@mae.cuhk.edu.hk

**Keywords:** microswimmer, biodegradable, magnetically powered, structural integrity

## Abstract

The propulsive efficiency and biodegradability of wireless microrobots play a significant role in facilitating promising biomedical applications. Mimicking biological matters is a promising way to improve the performance of microrobots. Among diverse locomotion strategies, undulatory propulsion shows remarkable efficiency and agility. This work proposes a novel magnetically powered and hydrogel-based biodegradable microswimmer. The microswimmer is fabricated integrally by 3D laser lithography based on two-photon polymerization from a biodegradable material and has a total length of 200 μm and a diameter of 8 μm. The designed microswimmer incorporates a novel design utilizing four rigid segments, each of which is connected to the succeeding segment by spring to achieve undulation, improving structural integrity as well as simplifying the fabrication process. Under an external oscillating magnetic field, the microswimmer with multiple rigid segments connected by flexible spring can achieve undulatory locomotion and move forward along with the directions guided by the external magnetic field in the low Reynolds number (Re) regime. In addition, experiments demonstrated that the microswimmer can be degraded successfully, which allows it to be safely applied in real-time in vivo environments. This design has great potential in future in vivo applications such as precision medicine, drug delivery, and diagnosis.

## 1. Introduction

Untethered mobile microrobots have demonstrated great potential in numerous microscale biomedical applications, such as minimally invasive therapy, drug and cell delivery, microsurgery, and in vivo sensing [1,2,3,4,5,6,7,8,9]. According to Purcell’s scallop theorem [10], in fluid environments with low Reynolds number (Re), viscous forces dominate compared to inertial forces. Thus, nonreciprocal motion is required to obtain net movement or displacement in low-Re Newtonian fluids. A wide range of remotely actuation methods has been used to actuate the movement of microrobots, such as magnetic fields, acoustic fields, lights, chemicals, and biohybrid strategies [7,9,11,12,13,14,15]. Magnetically driven strategies are more frequently used to actuate microrobots due to their advantages of precise controllability, driving capability, and safety [8,9,11,16,17,18].

Among various microrobots, microswimmers are a new form of cutting-edge technology that is designed to move in solutions and has the potential to provide a wide range of applications in medicine. Such technology can be in the form of either artificial microswimmers, robots which are engineered to swim and perform specific capabilities, or natural microswimmers such as bacteria and sperm cells [19]. In a general sense, they are simply microscopic-scale machines that are designed to perform and undergo specific motion and movement in response to external stimuli. Artificial magnetically torque-driven microswimmers come in a wide variety of shapes: helical [20,21], corkscrew [22], circular [23], and combinations of these shapes [24]. For any design, a key facet is ensuring that it can provide forward, precise, and controlled motion in various mediums. One advisable solution is adopting the efficient propulsion mechanism of body and caudal fin deformations to break the symmetry, hence achieving net propulsion in the low Re regime. Examples include fish-like multilink nanowire swimmers containing flexible porous Ag hinges [25], nanoswimmers made up of an elastic polypyrrole tail and flexible polymer bilayer hinges [26], multi-segment undulatory microswimmers containing joints and incorporating the use of a U-type transmission [27], sperm-shaped microswimmers made of flexible SU-8 or ultra-fine fiber tails [28,29], and so on. Besides, it is also highly important that the microrobots are made of a material that suits its usage and purpose, especially considering that many are used in complex environments such as in the human body [30]. This leads to the use of 3D-printed microswimmers with double-helical architecture which are hydrogel-based and enzymatically degradable [30,31], and superparamagnetic hydrogel swimming microrobots which allow a safe device degradation in vivo [32]. These biodegradable swimming microrobots move forward by mimicking the cork-screw propulsion in the low Reynolds number regime under a rotating magnetic field.

However, limitations do exist in this field. Most of these undulating microswimmers are fabricated from multiple materials, including soft components, to generate undulatory locomotion, complicating the manufacturing process of the microrobots [25,26,33]. In addition, structures with interfacing of rigid and soft parts are prone to repeated damage [34], increasing the likelihood of structural collapse during release and swimming. Although other undulatory microswimmers constructed by a single substrate may possess enhanced structural integrity, they lack biodegradability [27,28,29], which might create difficulties when applied in real-time in vivo environments as it can activate thrombi, potentially harming the organism. This limits its capabilities for use, especially in future applications within the human body. There is thus a notable demand to develop a new microswimmer with better rigidity, stronger structural integrity, and biodegradability, before the microswimmer can be readily applied to humans in future precision medicine.

The undulatory microswimmer proposed in this work employs a magnetically powered hydrogel-based biodegradable design. To achieve net propulsion in the low Re regime, the propulsion mechanism of body and caudal fin deformations is adopted in the design of a bioinspired travelling-wave microswimmer. The microswimmer utilizes four rigid segments, which are connected by soft springs to generate undulatory propulsion under an external oscillating magnetic field. It can be fabricated integrally from biodegradable materials using 3D laser lithography without further assembly, which enhances the structural integrity offered and allows for efficient and precise movement, as well as making it less susceptible to structural failure during movement. The main difference between this design and the previous biodegradable microswimmers reported in [30,31,32] is that the proposed microswimmer is with multi-segment architecture and actuated by an external oscillating magnetic field to move forward via undulatory propulsion, while the previous microswimmers [30,31,32] are with helical architectures and actuated by an external rotating magnetic field to move forward via cork-screw propulsion. The total length of the microswimmer is 200 μm, and it can be well controlled via external oscillating magnetic field to move forward along guided directions in low Re regime.

## 2. Materials and Methods

**Figure 1:**
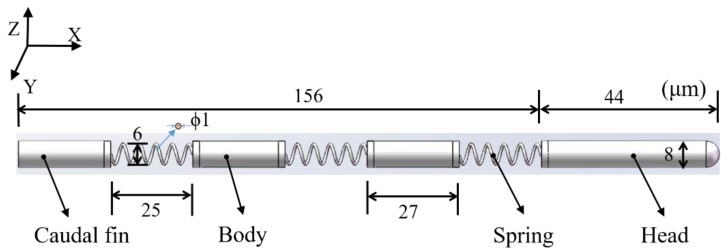
Schematic of the designed microswimmer.

Inspired by natural swimmers that swim with high efficiency and speed utilizing undulatory locomotion [25,26,27,28,29], the microswimmer was designed with multiple cylinders connected through springs to mimic the travelling-wave moment. The applied spring in the microswimmer can undergo flexible deformation under an external magnetic field to lead to the mechanical bending of the swimmer. The body and caudal fin in the design were used to break the symmetry in order to achieve net propulsion in the low Reynolds number environment. Under a periodic oscillating magnetic field, the magnetic head of the swimmer bends upward and downward regularly to achieve undulatory locomotion, propelling the swimmer forward. The overall length and diameter of the microswimmer are 200 and 8 μm, respectively, while the length, diameter, and wire diameter of the spring are 25, 6, and 1 μm, respectively, as shown in Figure 1 (a detailed justification of the design size of the microswimmer is provided in Appendix A). The head of the microswimmer is of a longer length compared with the other segments to obtain increased magnetization, creating a stronger magnetic torque exerted on the head [27]. The swimming direction of the microswimmer depends on the interaction between the head segment and the external magnetic field, where the head segment is magnetized along with the applied magnetic field. Therefore, the swimming direction can be altered by changing the direction of the magnetic field.

To be applied for in vivo applications, it is preferable the microswimmer be constructed using biodegradable materials to simultaneously avoid activation of immune system and vascular occlusion such as thrombi, as well as meet the required mechanical strength. Among the available biodegradable materials, poly(ethyleneglyco) diacrylatd (PEG-DA) is one of the most commonly used materials for biomedical applications, some of which are approved by Food and Drug Administration (FDA) for human use. As pure PEG-DA is too soft to form the microswimmer [32], the PEG-DA was combined with pentaerythritol triacrylate (PE-TA) to build the microswimmer, so that the microswimmer is biodegradable while still having sufficient mechanical strength. A small portion of superparamagnetic Fe_3_O_4_ nanoparticles are lastly added into the synthesized composite for magnetic actuation purpose. The microswimmer was fabricated by 3D laser lithography using a two photon write system (Nanoscribe), with an oil immersion objective of 63× NA1.4 (numerical aperture; GalvoScanMode). The 3D printing technology based on the two-photon polymerization principle enables the rapid manufacturing of geometrically-complex samples with nanoscale resolution [35]. The simple design, without complex connections, affords the option to undergo a simple fabrication process so that the designed microswimmer can be produced without requiring further assembly.

**Figure 2:**
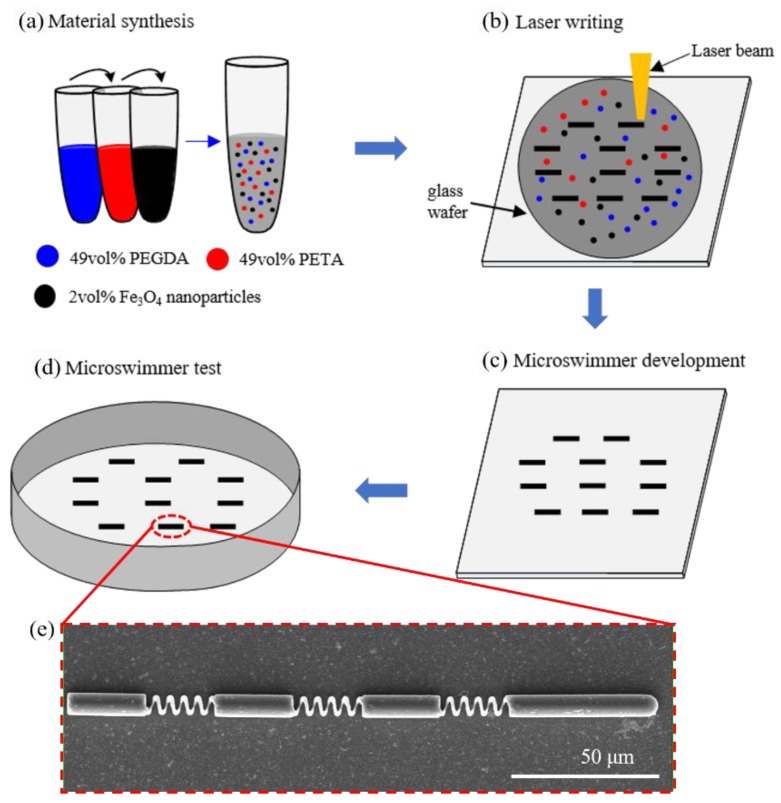
Fabrication process of the microswimmers: (**a**) material synthesis; (**b**) laser writing; (**c**) microswimmers development; (**d**) microswimmers test; and (**e**) scanning electron microscopy (SEM) image of the microswimmer.

Figure 2 shows the fabrication process of the microswimmer. The material preparation was first conducted, as shown in Figure 2a. The materials chosen are PEG-DA, a US FDA approved food-based polymer that has been shown to be safe for use inside the body, and PE-TA to increase mechanical strength [32]. These two materials allow the microswimmer to be biodegradable while still maintaining sufficient structural integrity. The optimum ratio used is 49% PEGDA and 49% PETA, together with superparamagnetic Fe_3_O_4_ nanoparticles blended in for magnetic actuation. Following the laser writing process, the prepared materials were dropped in a transparent glass wafer and the microswimmers directly printed by a laser beam, as shown in Figure 2b. After that, the glass wafer with the structures was fixed vertically in a 25 mL beaker filled with a bath of toluene substrate holder for 5 min to remove the unpolymerized photoresist, as shown in Figure 2c. Afterwards, the substrate holder was pulled out from toluene bath and placed in another beaker with iso-propanol for about 2 min. The substrate was then gently blown dry with nitrogen. Finally, Figure 2d shows that the prepared microswimmers were transferred to a chamber for swimming test. Figure 2e shows the scanning electron microscopy (SEM) images of the microswimmer. The efficiency of the release procedure for successful swimmers is more than 80%. Most of the loss occurred during the developing process, where chemical fluids used to remove unpolymerized photoresist may also remove parts of the structure.

## 3. Results and Discussion

**Figure 3:**
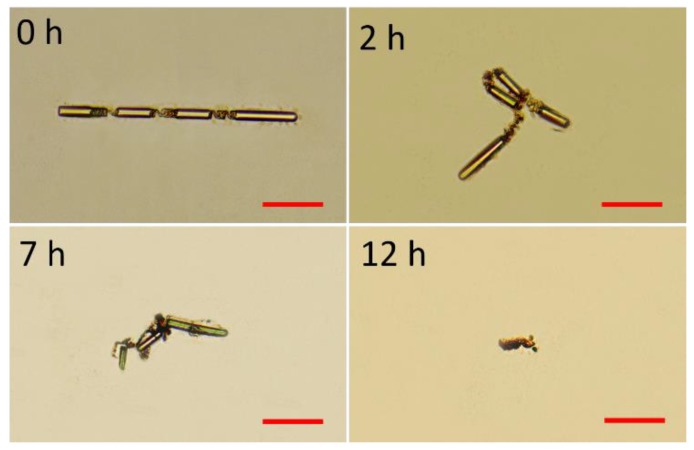
Degradability test of the fabricated microswimmer at different time instants. The scale bar is 50 μm for all images.

Degradability of the fabricated microswimmer was first tested. The components in the materials used to fabricate the swimmer have ester chemical bonds that could be slowly cleaved by water. As the components cleaved, the incorporated magnetic nanoparticles are released, and all the degraded product can be metabolized or circulated outside the body. To effectively demonstrate degradability of the microswimmers, the microswimmers were tested in sodium hydroxide (1 mol/L) of pH 14 environment for fast cleave of the chemical bonds. Aqueous sodium hydroxide was used to promote hydrolysis, which is also known as saponification [36]. The fabricated microswimmer was totally broken down in few hours, as shown in Figure 3, indicating that the microswimmers can be degraded in water environment for potential in vivo medical applications.

**Figure 4:**
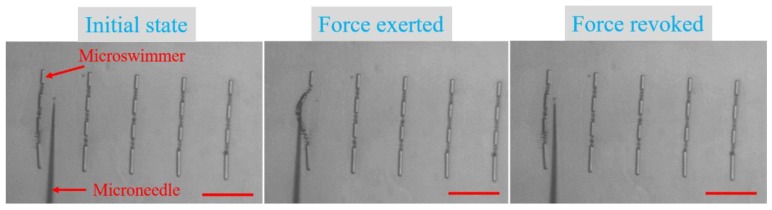
Tests of the structural integrity of the microswimmer. The scale bar is 100 μm for all images. Please refer to Video S1.

Maintaining a high level of structural integrity is a design objective of this microswimmer, hence the structural robustness test of the microswimmer was also conducted. A laboratory-designed microoperation system with a microneedle [37] was used to stir the microswimmer, which was adhered to the glass substrate. Force was applied to deform the middle sections connected with springs, and, when the force was revoked, the microswimmer was able to revert to its initial state, as shown in Figure 4 (Video S1). This test demonstrates that the microswimmer possesses the capacity of maintaining strong structural integrity, making structural collapse during operation unlikely. After all washing and release procedures were completed, microswimmers remained almost full integrity.

Free-swimming experiments of the microswimmer, where the microswimmer was detached by a microneedle and released into a water chamber, were performed to demonstrate its movement in the low Re regime under an external oscillating magnetic field. A magnetic actuation system [37] based on neodymium-iron-boron magnets and a DC motor was applied to drive the microswimmer by generating undulatory locomotion in x-y plane, propelling the microswimmer forward. Two neodymium-iron-boron magnets were used to generate a uniform magnetic field, where a DC motor controlled the oscillations of the magnets.

**Figure 5:**
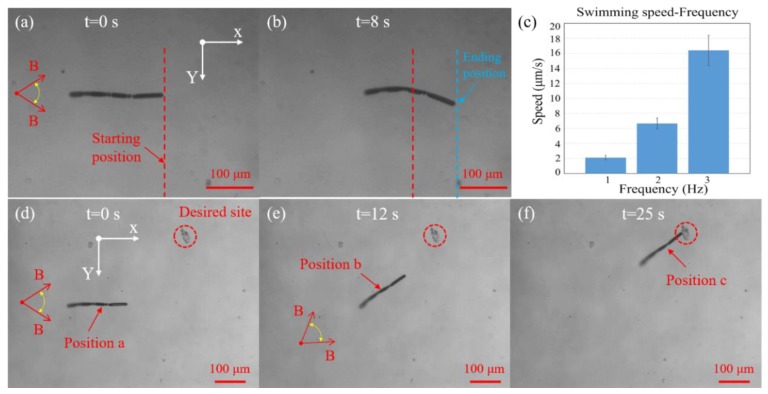
Free-swimming experiments of the microswimmer. (**a**,**b**) Net displacement of the microswimmer under an external oscillating magnetic field, where the oscillating frequency and amplitude of the magnetic field are 3 Hz and 45 degrees, respectively. The red dashed line indicates the starting position, and the blue dashed line indicates the ending position. Please refer to Video S2. (**c**) Microswimmer swimming speed against the frequency of the applied magnetic field. (**d**–**f**) Control the microswimmer to a desired site via external magnetic field. Please refer to Video S3.

Time-lapse images of the microswimmer position are illustrated in Figure 5a,b, where a magnetic field with a 3 Hz oscillating frequency and an amplitude of 45 degrees was applied for driving the microswimmer along positive x-axis (Video S2). This indicated that the microswimmer can achieve net displacement, with the swimming velocity measured approximately 16 μm/s. The forward undulatory locomotion of the microswimmer is attributed to the transfer of magnetic energy into periodic mechanical deformations of the microswimmer. This provides thrust, with the flexible spring structures providing the bending force needed to form undulatory locomotion. Period oscillation of the externally applied magnetic field is followed by the oscillation of the magnetized microswimmer head, which moves up and down, with the oscillations being transferred to the posterior segments, providing forward net displacement through travelling wave propulsion. The magnetic field frequency can be varied from 1 to 3 Hz using a DC motor, which allows controlling the velocity of travel. Figure 5c demonstrates the quantitative swimming velocity of the microswimmer against the frequency of applied magnetic field. It is seen that the microswimmer achieved faster swimming speed as the oscillating frequency increased from 1 to 3 Hz. Due to the drive limitation of the DC motor, higher oscillating frequencies cannot be provided.

Free-swimming experiments of guiding the microswimmer to a desired site through sub-paths of a–b and b–c was also conducted, as shown in Figure 5d–f (Video S3). At the beginning position a, the microswimmer was swimming forward along positive x-axis, before changing its swimming direction from horizontal to up-left direction at position b to agree with the direction of external magnetic field. Finally, the microswimmer was moving along up-left direction to the marked desired site via undulatory locomotion. This experiment indicated that the microswimmer can be well controlled along with guided directions via external magnetic field in the low Re regime. Free-swimming experiments of the microswimmers with other different sizes were also conducted, and the explanation and results are provided in Appendix A and Videos S4 and S5.

## 4. Conclusions

A magnetically powered and hydrogel-based biodegradable microswimmer was fabricated. The efficient propulsion mechanism of body and caudal fin deformations was adopted in the microswimmer design to achieve net displacements in the low Re regime. The microswimmer consisted of four rigid segments, each of which was connected to the succeeding segment by spring. The microswimmer was fabricated by 3D laser lithography from one base material without further assembly, simplifying the fabrication process while enhancing structural integrity offered and making it less susceptible to structural failure during movement. The microswimmer exhibited good degradability, which is advantageous to potential in vivo medical applications. The structural integrity tests of the microswimmer showed that the microswimmer was able to return to its initial state after disturbance, thus maintaining a strong structural robustness. Free-swimming experiments were conducted, indicating that the microswimmer was able to undergo net displacement with undulatory locomotion through the application of an external magnetic field, and the swimming direction of the microswimmer was well controlled.

## Figures and Tables

**Figure A1:**
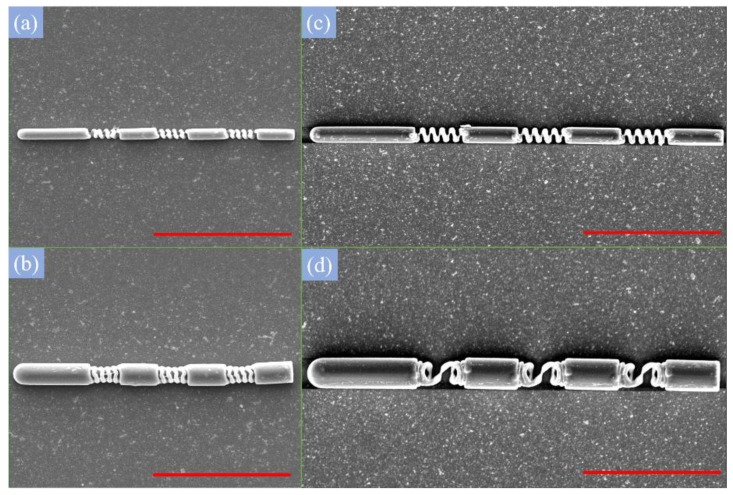
Scanning electron microscopy (SEM) images of the microswimmers with the diameter and the length of: (**a**) 4 and 100 μm; (**b**) 8 and 100 μm; (**c**) 6 and 150 μm; and (**d**) 12 and 150 μm. The scale bar is 50 μm for all images.

## Supplementary Materials

The following are available online at https://www.mdpi.com/2072-666X/11/4/404/s1, Video S1: Structural integrity tests of the microswimmer, Video S2: Net displacement of the microswimmer under an external oscillating magnetic field, Video S3: Free-swimming performance of guiding the microswimmer to a desired site through a sub-path, Video S4: Net displacement of the microswimmer with the diameter and the length of: (left) 4 and 100 μm; and (right) 8 and 100 μm, Video S5: Free-swimming performance of the microswimmer with the diameter and the length of 6 and 150 μm.

## Appendix A

In this work, the design aims of the microswimmer focus on two aspects: (1) the microswimmer can realize undulatory locomotion and achieve net displacement; and (2) the microswimmer can maintain a relatively small size. Due to the limitation of printing resolution, the wire diameter and the pitch of the spring cannot be too small, otherwise the spring will not be formed. In this work, based on the proposed printing materials (49 vol% PEG-DA, 49vol% PE-TA, 2 vol% Fe_3_O_4_ nanoparticles), the wire diameter and the pitch of the spring are required to be not less than 0.5 and 2.5 μm, respectively. The total length of the microswimmer is first determined as 100 μm to provide a template to design the microswimmer. The microswimmer consists of three springs, three body segments (including caudal segment), and a head. The rigid segments are required to be larger than the soft connection [25,26], which is the spring in this design. Therefore, the wire diameter and the pitch of the spring are selected to be 0.5 and 2.5 μm, respectively, and the coil number and the diameter of the spring are set as 5 and 3 μm, respectively. The length of the head of the microswimmer is selected to be 1.5 times the length of the rigid body segments [27]. Thus, the length of each body segment (including caudal segment) is calculated as approximately 14 μm, which is in accordance with the requirements between the rigid components and the soft connections.

Figure A1a shows the scanning electron microscopy (SEM) image of the microswimmer with the diameter and the length of 4 and 100 μm, respectively. In this case, no undulatory locomotion of the microswimmer was formed, and the net displacement can be neglected (Video S4). The microswimmer with the diameter and length of 8 and 100 μm, respectively, as shown in Figure A1b, was also tested in the free-swimming experiments, and the swimming result was similar to that with the microswimmer of diameter and length of 4 and 100 μm, respectively. A microswimmer which was scaled up 1.5 times (6 μm in diameter and 150 μm in length) based on the prototype microswimmer shown in Figure A1a was further tested, as shown in Figure A1c, and undulatory locomotion began to be exhibited in the free-swimming tests; nevertheless, the swimming speed was very low (Video S5). The spring of the microswimmer with the diameter and length of 12 and 150 μm, respectively, suffered from unexpected deformation, as shown in Figure A1d. When the prototype microswimmer was scaled up two times (8 μm in diameter and 200 μm in length), as presented in this work, it could realize undulatory locomotion as well as move forward favorably, while maintaining a small size.

## References

[1] Diller E., Sitti M. (2013). Micro-scale mobile robotics. Found. Trends Databases.

[2] Nelson B.J., Kaliakatsos I.K., Abbott J.J. (2010). Microrobots for Minimally Invasive Medicine. Annu. Rev. Biomed. Eng..

[3] Hu C., Pané S., Nelson B.J. (2018). Soft micro-and nanorobotics. Annu. Rev. Control Robot. Auton. Syst..

[4] Yan X., Zhou Q., Vincent M., Deng Y., Yu J., Xu J., Xu T., Tang T., Bian L., Wang Y.J. (2017). Multifunctional biohybrid magnetite microrobots for imaging-guided therapy. Sci. Robot..

[5] Jeon S., Kim S., Ha S., Lee S., Kim E., Kim S.Y., Park S.H., Jeon J.H., Kim S.W., Moon C. (2019). Magnetically actuated microrobots as a platform for stem cell transplantation. Sci. Robot..

[6] Li J., Li X., Luo T., Wang R., Liu C., Chen S., Li D., Yue J., Cheng S.-H., Sun D. (2018). Development of a magnetic microrobot for carrying and delivering targeted cells. Sci. Robot..

[7] Gao W., Wang J. (2014). Synthetic micro/nanomotors in drug delivery. Nanoscale.

[8] Peyer K.E., Zhang L., Nelson B.J. (2013). Bio-inspired magnetic swimming microrobots for biomedical applications. Nanoscale.

[9] Li J., de Ávila B.E.-F., Gao W., Zhang L., Wang J. (2017). Micro/nanorobots for biomedicine: Delivery, surgery, sensing, and detoxification. Sci. Robot..

[10] Purcell E.M. (1977). Life at low Reynolds number. Am. J. Phys..

[11] Ceylan H., Giltinan J., Kozielski K., Sitti M. (2017). Mobile microrobots for bioengineering applications. Lab Chip.

[12] Wang J., Gao W. (2012). Nano/microscale motors: Biomedical opportunities and challenges. ACS Nano.

[13] Gomez-Solano J.R., Bechinger C. (2015). Transient dynamics of a colloidal particle driven through a viscoelastic fluid. New J. Phys..

[14] Niu R., Fischer A., Palberg T., Speck T. (2018). Dynamics of binary active clusters driven by ion-exchange particles. ACS Nano.

[15] Choudhury U., Singh D.P., Qiu T., Fischer P. (2019). Chemical nanomotors at the gram scale form a dense active optorheological medium. Adv. Mater..

[16] Chen X.-Z., Hoop M., Mushtaq F., Siringil E., Hu C., Nelson B.J., Pané S. (2017). Recent developments in magnetically driven micro-and nanorobots. Appl. Mater. Today.

[17] Abbott J.J., Peyer K.E., Lagomarsino M.C., Zhang L., Dong L., Kaliakatsos I.K., Nelson B.J. (2009). How should microrobots swim?. Int. J. Robot. Res..

[18] Harduf Y., Jin D., Or Y., Zhang L. (2018). Nonlinear parametric excitation effect induces stability transitions in swimming direction of flexible superparamagnetic microswimmers. Soft Robot..

[19] Omori T., Ishikawa T. (2019). Swimming of spermatozoa in a maxwell fluid. Micromachines.

[20] Zhang L., Abbott J.J., Dong L., Kratochvil B.E., Bell D., Nelson B.J. (2009). Artificial bacterial flagella: Fabrication and magnetic control. Appl. Phys. Lett..

[21] Tottori S., Zhang L., Qiu F., Krawczyk K.K., Franco-Obregón A., Nelson B.J. (2012). Magnetic helical micromachines: Fabrication, controlled swimming, and cargo transport. Adv. Mater..

[22] Barbot A., Decanini D., Hwang G. (2016). On-chip microfluidic multimodal swimmer toward 3D navigation. Sci. Rep..

[23] Hou M.T., Shen H.-M., Jiang G.-L., Lu C.-N., Hsu I.-J., Yeh J.A. (2010). A rolling locomotion method for untethered magnetic microrobots. Appl. Phys. Lett..

[24] Cappelleri D.J., Bi C., Guix M. (2018). Tumbling Microrobots for Future Medicine: Robots smaller than a grain of sand could move through the body by tumbling end over end, opening up the possibility of intriguing biomedical applications. Am. Sci..

[25] Li T., Li J., Zhang H., Chang X., Song W., Hu Y., Shao G., Sandraz E., Zhang G., Li L. (2016). Magnetically propelled fish-like nanoswimmers. Small.

[26] Jang B., Gutman E., Stucki N., Seitz B.F., Wendel-García P.D., Newton T., Pokki J., Ergeneman O., Pané S., Or Y. (2015). Undulatory locomotion of magnetic multilink nanoswimmers. Nano Lett..

[27] Liao P., Xing L., Zhang S., Sun D. (2019). Magnetically driven undulatory microswimmers integrating multiple rigid segments. Small.

[28] Khalil I.S., Dijkslag H.C., Abelmann L., Misra S. (2014). MagnetoSperm: A microrobot that navigates using weak magnetic fields. Appl. Phys. Lett..

[29] Khalil I.S., Tabak A.F., Hosney A., Mohamed A., Klingner A., Ghoneima M., Sitti M. Sperm-shaped magnetic microrobots: Fabrication using electrospinning, modeling, and characterization. Proceedings of the 2016 IEEE International Conference on Robotics and Automation (ICRA).

[30] Ceylan H., Yasa I.C., Yasa O., Tabak A.F., Giltinan J., Sitti M. (2019). 3D-printed biodegradable microswimmer for theranostic cargo delivery and release. ACS Nano.

[31] Wang X., Qin X.H., Hu C., Terzopoulou A., Chen X.Z., Huang T.Y., Maniura-Weber K., Pané S., Nelson B.J. (2018). 3D printed enzymatically biodegradable soft helical microswimmers. Adv. Funct. Mater..

[32] Peters C., Hoop M., Pané S., Nelson B.J., Hierold C. (2016). Degradable magnetic composites for minimally invasive interventions: Device fabrication, targeted drug delivery, and cytotoxicity tests. Adv. Mater..

[33] Tottori S., Nelson B.J. (2018). Controlled propulsion of two-dimensional microswimmers in a precessing magnetic field. Small.

[34] Bartlett N.W., Tolley M.T., Overvelde J.T., Weaver J.C., Mosadegh B., Bertoldi K., Whitesides G.M., Wood R.J. (2015). 3D-printed, functionally graded soft robot powered by combustion. Science.

[35] Cumpston B.H., Ananthavel S.P., Barlow S., Dyer D.L., Ehrlich J.E., Erskine L.L., Heikal A.A., Kuebler S.M., Lee I.-Y.S., McCord-Maughon D. (1999). Two-photon polymerization initiators for three-dimensional optical data storage and microfabrication. Nature.

[36] Browning M.B., Cosgriff-Hernandez E. (2012). Development of a biostable replacement for PEGDA hydrogels. Biomacromolecules.

[37] Liao P., Li J., Zhang S., Sun D. A fish-like magnetically propelled microswimmer fabricated by 3D laser lithography. Proceedings of the IEEE International Conference on Robotics and Automation (ICRA).

